# Intradermal Allergen Immunotherapy for Allergic Rhinitis: Current Evidence

**DOI:** 10.3390/jpm12081341

**Published:** 2022-08-21

**Authors:** Kawita Atipas, Dichapong Kanjanawasee, Pongsakorn Tantilipikorn

**Affiliations:** 1 Division of Rhinology and Allergy, Department of Otorhinolaryngology, Faculty of Medicine Siriraj Hospital, Mahidol University, Bangkok 10700, Thailand; 2 Center of Research Excellence in Allergy & Immunology, Faculty of Medicine Siriraj Hospital, Mahidol University, Bangkok 10700, Thailand; 3 Biodesign Innovation Center, Department of Parasitology, Faculty of Medicine Siriraj Hospital, Mahidol University, Bangkok 10700, Thailand

**Keywords:** allergic rhinitis, allergen immunotherapy, intradermal, intradermal immunotherapy, alternatives

## Abstract

Allergic rhinitis (AR) is an immunoglobulin E (IgE)-mediated inflammatory disease that is induced by allergen introduction to the nasal mucosa, which triggers an inflammatory response. The current treatments for AR include allergen avoidance and pharmacotherapy; however, allergen-specific immunotherapy (AIT) is the only treatment that can be employed to modify immunologic responses and to achieve a cure for allergic diseases. The current standard routes of AIT administration are the subcutaneous and sublingual routes. Alternatively, the dermis contains a high density of dermal dendritic cells that act as antigen-presenting cells, so intradermal administration may confer added advantages and increase the efficacy of AIT. Moreover, intradermal immunotherapy (IDIT) may facilitate a reduction in the allergen dosage and a shortening of the treatment duration. The aim of this review was to search and evaluate the current evidence specific to IDIT, including its modified formulations, such as allergoids and peptides. The results of this review reveal conflicting evidence that suggests that the overall benefit of IDIT remains unclear. As such, further clinical trials are needed to establish the clinical utility of IDIT, and to determine the optimal treatment-related protocols.

## 1. Introduction

Allergic rhinitis (AR) is an immunoglobulin E (IgE)-mediated inflammatory disease that is induced by the inhalation of airborne allergens by sensitized individuals [1,2]. AR is a common global health problem that affects 10–40% of the population [3]. AR is associated with a high burden of disease, and it greatly impairs general and disease-specific quality of life, sleep quality, and daily function [1,4]. Thus, treatment and prevention modalities, such as allergen avoidance, pharmacotherapy, and allergen-specific immunotherapy (AIT), are important for improving both symptoms and patient well-being.

However, unlike allergen avoidance and pharmacotherapy, where the aim of therapy is to reduce symptoms, but the disease trajectory is not affected, AIT remains the only known method for modifying immunologic responses in IgE-mediated allergic disease. AIT is an immunomodulating process that is induced by the administration of a specific allergen to the level of desensitization and immunotolerance [5]. This method has been long accepted as an effective tool for ameliorating allergic symptoms, slowing disease progression, and preventing new sensitization. AIT confers long-term clinical benefits that may persist for years after treatment discontinuation [1,6,7,8,9].

The first study of AIT was conducted in 1910 by Noon and colleagues. The study involved subcutaneous inoculation of a pollen extract in patients with hay fever, and the treatment yielded favorable results [10,11]. Since then, AIT has been intensively researched and has become a standard of care for patients with AR.

The goal of AIT is to target the immune system efficiently and safely via lymphatic organs, and the current standard routes of administration of immunotherapy are the subcutaneous (SCIT) and sublingual (SLIT) routes. Systemic reviews and meta-analyses showed that both SCIT and SLIT are effective in reduction of symptoms and medication use, with an acceptable safety profile [7,8,12]. However, these approaches involve some minor drawbacks, such as prolonged treatment duration, complex dosing adjustments, and high cost of treatment [13]. The incidence of therapeutic withdrawal is quite high for both methods [14]. However, there is a low risk of severe systemic side effects, especially for SCIT, which has a reported rate of anaphylaxis of 2.1% [15].

In an attempt to improve efficacy, safety, duration of treatment, and dosage volume, other alternative routes of administration, including intralymphatic immunotherapy (ILIT), epicutaneous immunotherapy (EPIT), intranasal immunotherapy (INIT), and intradermal immunotherapy (IDIT), have been proposed. Of these, the transcutaneous options (SCIT, IDIT, or EPIT) are considered to be the most dependable routes of administration for AIT, as well as for vaccination. In addition to the skin being the largest organ of the human body, it is underlaid by an abundance of immune cells and lymphatic channels, which makes it an ideal organ system through which therapies designed to influence immunity modulation and immunization can be administered.

Since the establishment of SCIT as the most accepted skin-related route of administration due to its well-documented safety and efficacy, other skin-related methods for providing immunotherapy have been insufficiently investigated. SCIT triggers immunomodulation by targeting antigen-presenting cells (APC) located beneath the skin surface; however, countless APCs are actually located in the skin’s dermis. Thus, when compared to conventional SCIT, IDIT has—at least in theory—the potential of being a comparatively more favorable immunomodulatory route of administration. The intradermal route of administration in this clinical setting has thus far been underexplored, and its use has been overshadowed by other therapeutic options. Accordingly, the aim of this review was to search the published literature, and to evaluate the current evidence specific to IDIT and its potential as an alternative to the conventional method of AIT.

## 2. Intradermal Vaccination

Vaccination through the intradermal route has been approved for several vaccines, such as rabies, influenza, and Bacillus Calmette–Guérin (BCG), and due to the potential for a reduced dose of administration, it is currently under investigation as a potential route for other vaccines [16]. The most prominent of these is the COVID-19 vaccine [17,18]. The dermal dendritic cells (DCs) and epidermal Langerhans’ cells (LCs) exist in abundance in the skin and play a crucial role in antigen presentation. After encountering the antigens, these cells migrate to lymphatic nodes and stimulate immune responses [19,20].

A recent systematic review by Schnyder et al. [21] compared reduced dosages of antigen vaccination among intradermal, intramuscular, and subcutaneous injection. They found fractional doses of influenza, rabies, and hepatitis B given intradermally to be effective and potentially superior to other routes of vaccine administration. That group also reported comparable side effects among the three routes. However, and not surprisingly, the number of minor local adverse events that they reported was higher in the intradermal delivery group.

## 3. Intradermal Immunotherapy (IDIT): Pathophysiology and Mechanism

**Figure 1:**
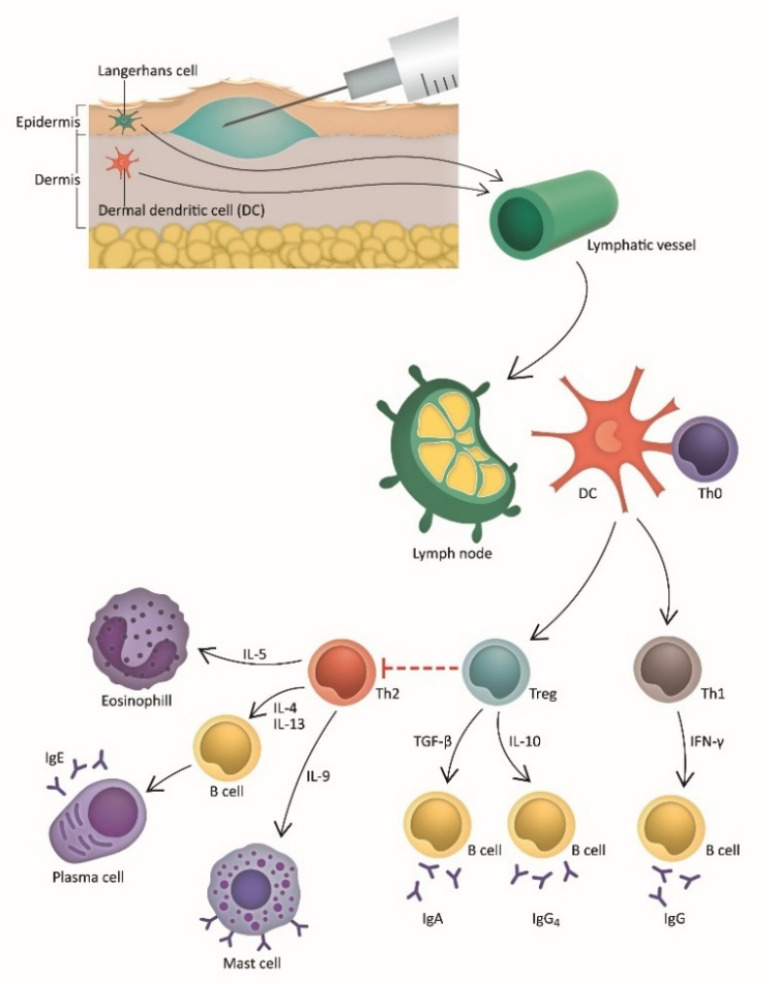
The mechanism of intradermal immunotherapy (IDIT). Abbreviations: DC, dendritic cells; Ig, immunoglobulin; IL, interleukin; Th, T helper cells; Treg, regulatory T cells.

The dermis contains a high density of dermal dendritic cells (DCs), which are antigen-presenting cells. Allergens that enter the body through damaged skin are carried by dermal DCs and can induce proinflammatory responses [22]. The trigger of regulatory T cell and B cell-mediated immune response leads to a shift in the immune system, from a predominantly T-helper 2 (T_H_2) cytokine response to that of T-helper 1 (T_H_1). This influences the release of transforming growth factor beta (TGF-β), interferon gamma (IFN-γ), and interleukin 10 (IL-10). Consequently, the induction of allergen-specific immune tolerance is achieved [23]. Additionally, Treg cells influence a reduction in IgE and enhance the release of IgG4/IgA-blocking antibodies [24,25,26]. The mechanism of IDIT is pictorially demonstrated in Figure 1. The ability of DCs to migrate through lymphatic vessels to skin-draining lymph nodes and induce Treg cells suggests IDIT is a promising route of administration [27,28]. As evidenced by other vaccines, the higher number of DCs in the dermis theoretically induces higher immunogenicity when compared to SCIT [16,21].

The aforementioned immunologic response profile was previously demonstrated in a mouse model compared to other routes of administration. Intradermal delivery of the antigen was shown to elevate specific IgG production and lower IgE production in a way that was superior to that observed after subcutaneous injections or after the use of epicutaneous patches [29]. Moreover, repeated doses of intradermal injection were shown to be capable of inhibiting ongoing IgE production in high serum IgE-induced mice [29].

## 4. Clinical Studies of Intradermal Immunotherapy

In 1926, Phillips [30] reported a study of 29 patients with hay fever who received intradermal injections of pollen extracts. All patients experienced early relief of symptoms, and the treatment was concluded to be safe. Seven years later, Phillips [31] published a larger study of 322 patients with 91.6 percent reporting subjective satisfactory relief of symptoms. Only 12 general reactions occurred, and one subject required adrenaline. The investigators described their support of the intradermal route of antigen administration due to the lower dosage required and the prompt relief of symptoms.

In 2012, Rotiroti et al. [32] conducted a study in adults with dual sensitization to timothy grass (*Phleum pratense*) and silver birch pollen (*Betula verrucosa*) to evaluate the effect of low-dose intradermal injections of timothy grass extract (estimated 7 ng of Phl p5) given at enrollment, followed by every 2 weeks for 10 weeks. Controls received concomitant grass and birch pollen extract given at week 0 and 10 or a single injection at week 10. The result demonstrates the suppression of the cutaneous response 24 h after IDIT for grass pollen in the group that received repeated doses compared to both control groups. The immunologic study showed an increase in specific IgG antibodies and an increase in the blockade of allergen–IgE binding to B cells in the repeated injection group. A fold increase in IgG1 was also observed, but the increases in the absolute levels of specific IgG1 and IgG4 were not statistically significant. Patient symptoms were not described in that study.

Following the publishing of a pilot study by Hernández et al. [33] that reported a reduction in the total nasal symptom score (TNSS) and the face visual analog scale (fVAS) in eight children with allergic rhinitis treated with IDIT, Rondon et al. [34] recently published a study on IDIT in children diagnosed with perennial allergic rhinitis and a house dust mite (HDM) allergy. Interestingly, instead of using the usual allergen extracts, the authors prepared a flask containing a mixture of *Dermatophagoides pteronyssinus*/*Dermatophagoides farinae* and *Blomia tropicalis* and injected 0.05 mL of the mixture intradermally (approximately 8 AU of *Dermatophagoides pteronyssinus*/*Dermatophagoides farinae* and 0.12 mcg of *Blomia tropicalis*). Patients then received this same volume at increasing intervals for one year. The result among the 17 patients who completed the AIT course shows an improvement in both the TNSS and the fVAS. In addition, IgG4 and IL-10 levels both increased. It should be noted that the Rondon et al. study did not include a control group.

Despite the congruence of evidence thus far regarding the safety and efficacy of intradermal immunotherapy, one study has reported contrary findings. In one of the largest clinical trials on IDIT by Slovick et al. [35] in 2016, 93 adults with timothy grass (*P. pratense*)-induced allergic rhinitis were randomized to receive either seven intradermal injections of *P. pratense* extract (7 ng of Phl p5) or a histamine placebo pre-seasonally. The results of that study show the combined symptom and medication scores (CSMS), the overall symptom scores, and the amount of rescue medication used not to be significantly different between the AIT group and the placebo/control group during the grass pollen season. Moreover, and interestingly, in per-protocol analysis, the treatment group had worse nasal symptoms and fewer symptom-free days compared to the placebo group. Furthermore, the immunologic study showed a significant decrease in IgE in the control group, but not in the treatment group. Similarly, skin biopsies revealed higher T_H_2 markers and lower T_H_1 markers in the treatment group. The data are summarized in Table 1.

## 5. Modified Formulations of Intradermal Immunotherapy

### 5.1. Allergoids

Allergoids are modified allergen extracts whose allergenicity has been reduced with preserved immunogenic activity [36]. Allergoids are commonly administered subcutaneously; however, Martinez et al. [37] conducted the first randomized controlled trial (RCT) in IDIT using *P. pratense* allergoid with a satisfactory result. Subjects were randomized to receive six intradermal weekly injections of placebo, low-dose allergoid, or high-dose allergoid. Patients receiving a high dose (0.06 ug protein/dose) reported a better CSMS when compared to the placebo group. A conjunctival provocation test after the first pollen season revealed that a higher concentration was needed to induce a reaction in both treatment groups. IgE levels varied but specific IgEs for *P. pratense* decreased after the second year of treatment in the high-dose group compared to the levels observed at baseline and the first year. IgG4 levels did not increase; however, the authors suggested that this could be due to the timing of the test (Table 2).

### 5.2. Peptide Immunotherapy

Synthetic peptide immunoregulatory epitope was developed to induce T cell tolerance while reducing IgE cross-linking [38]. Peptide immunotherapy was administered subcutaneously in the early studies [39,40]; however, several subsequent trials were con-ducted using the intradermal route. An RCT by Ellis et al. [41] in 2017 in grass allergen peptides administered intradermally demonstrated improvement in symptoms compared to the placebo when subjects were challenged in the environmental exposure unit (EEU). A follow-up study 2 years after treatment continued to show a trend toward symptom improvement; however, the result was not statistically significant [42].

Several trials in cat allergen (Fel d 1) peptides administered intradermally were also found in the published literature. Although many of those trials were conducted in asthmatic subjects, some studies were conducted in allergic rhinitis patients. A study by Worm et al. [43] demonstrated the safety of Fel d 1 peptide immunotherapy in both intradermal and subcutaneous groups. That study reported a non-significant trend toward a reduction in late-phase skin reactions (LPSR) when peptides were administered intradermally. Patel et al. [44] reported a reduction in the total rhinoconjunctivitis symptom score (TRSS) using the environmental exposure chamber challenge in subjects receiving four administrations of 6 nmol over 12 weeks compared to the 3 nmol regimen and placebo at the 1-year follow up. A follow-up study by that same group confirmed persistent benefits for as long as 2 years [45], and no serious adverse event was observed. However, the following unpublished phase III study of Fel d 1 allergen peptides had a large placebo effect, in which the intradermal therapy could not demonstrate benefits over that of the placebo. The data are summarized in Table 3.

## 6. Discussion

This review included studies in AIT and its modified forms given via the intradermal route. Although intradermal injection has long been utilized for vaccination, its application to immunotherapy is comparatively limited. The target of intradermal injection is the papillary dermis, which has a high density of dendritic cells [20]. As such, and as demonstrated in several immunologic studies of various intradermal vaccines, intradermal injection can be expected to induce an immune response with allergen dosages that are 1/10 or 1/5 of the dosages needed when administered via the SC or IM routes [17].

Until now, studies in intradermal allergen immunotherapy are limited and there is a lack of controlled clinical trials. However, among the studies, some conflicting evidence has been reported. Three observational studies, two of which were published by Phillips in the early 20th century [30,31], and a recent study by Rondon et al. [34], suggested the benefit of IDIT relative to symptom improvement. However, those three studies did not include control subjects, which makes it impossible to exclude the possibilities of a placebo effect. Among the reported studies, a preliminary study showed that IDIT for grass pollen could suppress cutaneous late response; however, a follow-up study by Slovick et al. [35] that included a placebo-controlled group demonstrated neither clinical nor immunological improvement. In fact, subjects in the treatment group experienced an overall higher symptom score, an increase in the level of specific IgE antibody, and higher levels of T_H_2 marker.

There are some potential reasons that may explain the discrepancies in published results. First, there is not yet an agreed upon and widely accepted standard dosage for ID-IT. Anecdotally, it is well-accepted that IDIT requires a lower allergen dosage; however, the standard effective dose remains unestablished. The allergen dosages used in the included studies varied from 10–100 times less than the effective dose used in SCIT. Details specific to the allergen dosage(s) used in each of the included studies are presented in Table 1. Second, the duration of treatment varied from study to study. The one high-quality clinical trial by Slovick et al. [35] administered IDIT over a 12-week duration, while others ranged from as short as 10 weeks to as long as 1 year. Since an initial AIT treatment duration of at least 3 years is recommended in guidelines for long-term benefits [15,46], the duration of IDIT in some of the included studies would be considered too short to yield any demonstrable benefits. Third, as mentioned by Slovick et al. [35], the reported deterioration of clinical symptoms in some studies may have been due to the priming effect. Lastly, variation in the types of allergens used for IDIT across studies may have contributed to the inconsistency of results, since there is currently no method to standardize the different responses to AIT between the different indoor and outdoor allergens. Therefore, it may be premature to conclude that IDIT is a less effective modality for treating allergic rhinitis.

Moreover, some of the major concerns regarding the efficacy and side effects of IDIT have been addressed via the use of adjuvants and improvements in allergen characteristics. Modifications of typically allergenic proteins to become hypoallergenic, such as allergoids, recombinant peptides, and non-IgE peptides, should theoretically cause fewer side effects and allow administration of higher allergen doses.

In addition, modified formulations of IDIT have shown more promising results than crude extracts. A placebo-controlled study of subjects who were given the *P. pratense* allergoid intradermally achieved better symptom control than in the control group [37]. The results from studies that investigated peptide immunotherapy are more controversial. A placebo-controlled study of grass allergen peptides given intradermally showed benefits in symptom control for some, but not all, dosing regimens [41,42]. Most studies of cat peptides did not show statistically significant effects, with a phase III study failing to demonstrate any benefit over the placebo [44,45].

Overall, the benefit of IDIT relative to dose reduction and enhancing immunogenicity remains unclear. Although the evidence from previous studies does seem to affirm the general safety of IDIT with no major adverse events, the number of studies that focused sufficiently on different allergen types, dosages, and dosing intervals are insufficient. Moreover, no direct head-to-head comparison with SCIT and SLIT regarding efficacy and safety has so far been reported and further clinical trials are required. Another transcutaneous route of administration is EPIT, in which skin is treated by methods such as abrasion, adhesive tape striping, or microneedle to enhance allergen penetration to epidermal LCs. Similarly, no comparison with IDIT has been reported.

Modified formulations may show more therapeutic potential, but there remain many controversies. There is a need for further evidence, especially regarding the validation of dosages and dosing techniques. A major disadvantage of IDIT is that it still requires injections, which does little to assuage the fears of those suffering from trypanophobia. In addition, the intradermal route is a more technically demanding route of administration, it is associated with a higher rate of local reaction, and it potentially causes more discomfort [47]. Ultimately, although there is real potential for reduction in the dosage and therefore the cost, the need to modify products to make them safer and more effective would nullify any foreseeable cost-saving benefit in resource-limited settings.

## 7. Conclusions

The results of this review reveal the benefits of IDIT to be inconclusive, with conflicting findings among published studies and inadequate evidentiary support from high-quality trials. By way of example, some studies reported improvement in clinical symptoms and biomarkers; however, one major RCT demonstrated a negative result in the treatment group compared to the placebo control.

Despite an attempt to enhance specificity by modifying the injected allergenic com-ponents, IDIT’s benefits still remain unclear. Nevertheless, given all the potential benefits of IDIT, it remains prudent to keep IDIT as a viable treatment option. Further clinical trials are required to establish stronger evidence of benefits and to determine the optimal allergen administration protocols.

## Figures and Tables

**Table 1:** Clinical studies of IDIT.

Study	Study Design	Allergen	Study Population	Dose	Duration	Outcome Measure	Results
Phillips et al. (1926) [30]	Cohort	Local pollens	NR (*n* = 29)	-Injection with increasing dose with 3 or 6 doses/week adjusted by patient’s size of local sensitization. -Interval was increased after the relief of symptoms.	NR	-Relief of symptoms -Safety	-Complete or near relief occurred in all 29 cases. -Symptom relief was shown to be associated with the size of local reaction.
Phillips et al. (1933) [31]	Cohort	Local pollens	Children and adults (*n* = 322)	-Injection with increasing dose daily, adjusted according to local reaction. -Interval was increased after the relief of symptoms.	NR	-Relief of symptoms -Safety	-91.6% of the patients expressed satisfactory relief. -Prompt relief was usually before 7 days. -12 general reactions occurred in nine patients (1: 625 doses). -There was no fatal reaction.
Rotiroti et al. (2012) [32]	RCT	Timothy grass (*Phleum pratense*) and/or silver birch (*Betula verrucosa*) pollen	Adults (10/10/9) (*n* = 29)	Injection of 0.1, 1.0, 10 BU of grass and/or birch pollens each visit; 2-week interval/visit Group A: Grass and birch pollen extracts at visits 1 and 6, grass pollen extract at visits 2–5; Group B: Grass and birch pollen extracts at visits 1 and 6; Group C: Grass and birch pollen extracts at visit 6 only	10 weeks	-Cutaneous response -Specific IgG, IgG1, IgG4, IgE-FAB	-Cutaneous response at 24 h was significantly suppressed in group A compared to groups B and C, although early responses were equivalent among groups. -Grass pollen-specific IgG was increased in group A at both week 6 and week 10. -Absolute level of IgG1 and IgG4 were not statistically increased; however, 2.4-fold increase of IgG1 was observed in group A. -Increase in inhibition of IgE allergen complex binding to B cells in group A between week 6 and week 10.
Slovick et al. (2016) [35]	RCT	Timothy grass (*Phleum pratense*) pollen	Adults (47/46) (*n* = 93)	Injection of 10 BU of grass pollen or histamine pre-seasonally for 7 visits at 2-week intervals Group A: grass pollen Group B: histamine	12 weeks	-CSMS -Symptoms score -Medication score -VAS -Mini-RQLQ -EQ-5D-5L -Medication/symptom-free day -AEs -Skin biopsy -Cutaneous response -Serum-specific IgE, IgG -Basophil activation test	-CSMS was similar between two groups over the entire pollen season. -Nasal symptom score and VAS nasal score were 44% and 28% higher, respectively, in the treatment group. -Mini-RQLQ scores, EQ-5D-5L scores, and the numbers of symptom-free or medication-free days were not different between both comparisons. -There were no serious AEs. -There was no difference in AEs between both comparisons. -Skin surface markers demonstrated higher T_H_2 marker CRTH2 expression and lower T_H_1 cell marker CXCR3 in treatment group. -Suppression of late-phase cutaneous response was shown at 4 and 7 months but not at 10 and 13 months. -Serum-specific IgE reduction was lower in the treatment group. -Serum-specific IgG4 was similar between both comparisons. -There was no significant effect of treatment on basophil activation markers.
Vieira-Hernández et al. (2018) [33]	Cohort ; A pilot study of Rondon et al. (2021) [34]	Mixed dust mite (*Dermatophagoides pteronyssinus*/*Dermatophagoides farinae* and *Blomia tropicalis*) -5 ng of HDM major allergens and 2.5 DBU of *Blomia tropicalis* allergens per 0.05 mL.	Children (*n* = 8)	Injection of allergen with a mix of 5 ng of *Dermatophagoides pteronyssinus/Dermatophagoides farinae* and 2.5 DBU of *Blomia tropicalis* at a 1-week interval x 3 months	12 weeks	-TNSS -fVAS -Serum-specific IgG4	-TNSS and fVAS were decreased. -Specific IgG4 was significantly increased for *Blomia tropicalis*, and a trend toward increased specific IgG4 was observed for *Dermatophagoides pteronyssinus/Dermatophagoides farinae*.
Rondon et al. (2021) [34]	Cohort	Mixed dust mite (*Dermatophagoides pteronyssinus/Dermatophagoides farinae* and *Blomia tropicalis*)	Children (*n* = 17)	Injection of allergen with a mix of 50 ng of *Dermatophagoides pteronyssinus/Dermatophagoides farinae* and 120 ng of *Blomia tropicalis* at a 1-week interval x 3 months, followed by a 2-week interval x 3 months, followed by a 3 week interval x 3 months, followed by a 4-week interval x 3 months	1 year	-TNSS -fVAS -Serum-specific IgE, IgG4, IL 10	-TNSS and fVAS were decreased after 42 and 49 days, and remained so until 1 year. -Specific IgG4 and IL-10 were increased after treatment. -Only minor local reactions were observed.

Abbreviations: AEs, adverse events; BU, biological unit; CSMS, combined symptom and medication score; DBU, diagnostic biological unit; EQ-5D-5L, EuroQol 5-Dimensions and 5 Levels of Severity General Health State Survey; fVAS, face visual analog scale; HDM, house dust mite; IgE-FAB, IgE-dependent facilitated allergen binding; IL, interleukin; Mini-RQLQ, Mini Rhinitis Quality of Life Questionnaire score; NR, not reported; Ig, immunoglobulin; RCT, randomized controlled trial; TNSS, Total Nasal Symptom Score; VAS, visual analogue scale.

**Table 2:** Clinical studies of allergoids.

Study	Study Design	Allergen	Study Population	Dose	Duration	Outcome Measure	Results
Martínez et al. (2020) [37]	RCT	Timothy grass (*Phleum pratense*) allergoid	Children and adults (53/42/53) (*n* = 148)	Injection of allergoid 0.03 or 0.06 μg protein/dose or placebo pre-seasonally for 2 consecutive years at 1-week intervals for 6 weeks each year Group A: low dose Group B: high dose Group C: placebo	2 years	-CSMS -Symptom score -Medication score -Medication/symptom-free day -Conjunctival provocation test -Serum-specific IgE, IgG4 -AEs	-High-dose group had lower CSMS than the low-dose or placebo groups. -Increase in protein concentrations was needed to induce the conjunctival provocation test in all active groups. -*P. pratense* IgE level after 2 years in high-dose group was lower than baseline. -Specific IgG4 level did not increase in any group. -There were no differences in AEs between the treatment and placebo group.

Abbreviations: AEs, adverse events; CSMS, combined symptom and medication score; Ig, immunoglobulin; RCT, randomized controlled trial.

**Table 3:** Clinical studies of peptide immunotherapy.

Study	Study Design	Allergen	Study Population	Dose	Duration	Outcome Measure	Results
Ellis et al. (2017) [41]	RCT	Mixed grass allergen peptides (derived from Cyn d 1, Lol p 5, Dac g 5, Hol l 5, and Phl p 5)	Adults (71/70/71/70) (*n* = 282)	Injection of allergen peptide at different intervals pre-seasonally Group A: 6 nmol pep-tide at 2-week intervals for 8 doses Group B: 12 nmol peptide at 4-week intervals for 4 doses Group C: 12 nmol peptide at 2-week intervals for 8 doses Group D: placebo	14 weeks	-TRSS (4 days of EEU challenge at 25 weeks post-treatment initiation) -Serum-specific IgA, IgE, and IgG4 -AEs	-The mean TRSS was significantly improved only in group A compared to the placebo. -Group B showed a reduction in TRSS at all but one time point after the EEU challenge compared to placebo. -TRSS in group C was not different from that of the placebo. -There were no significant changes in specific IgA, IgE, and IgG4 levels from baseline in all groups. -There was a similar rate of AEs between the treatment and placebo group.
Ellis et al. (2020) [42]	RCT; Follow-up study of Ellis et al. (2017)	Mixed grass allergen peptides (derived from Cyn d 1, Lol p 5, Dac g 5, Hol l 5, and Phl p 5)	Adults (1-year, *n*= 122; 2 year, *n* = 85)	Injection of allergen peptide in different interval pre-seasonally Group A: 6 nmol peptide at 2-week intervals for 8 doses Group B: 12 nmol peptide at 4-week intervals for 4 doses Group C: 12 nmol peptide at 2-week intervals for 8 doses Group D: placebo	14 weeks	-TRSS (4 days of EEU challenge at 1 year and 2 years post-treatment initiation) -Serum-specific IgA, IgE, and IgG4 -AEs	-Group A and B regimens demonstrated a trend toward a reduction in the mean TRSS after the EEU challenge compared to the placebo at 1-year and 2-year follow ups. However, no statistical significance was shown. -Group C showed effects comparable to that of the placebo. -There were no changes in specific IgA, IgE, and IgG4 levels from the baseline in all groups.
Worm et al. (2011) [43]	RCT	Cat allergen peptide (derived from Fel d 1)	Adults (40/48) (*n* = 88)	Single injection of allergen peptide at different concentrations or placebo Group A: IDIT (dose level of 0.03, 0.3, 1, 3, 12 nmol, placebo) Group B: SCIT (dose level of 0.03, 0.3, 1, 3, 12, 20 nmol, placebo)	Single injection	-Cutaneous response -AEs	-IDIT showed a trend toward a reduction in cutaneous response at 8-h, 21 days after injection with a 3 nmol dose compared to the placebo; however, no statistical significance was shown. -There was no severe AE reported.
Patel et al. (2013) [44]	RCT	Cat allergen peptide (derived from Fel d 1)	Adults (67/66/69) (*n* = 202)	Injection of allergen peptide at different intervals Group A: 3 nmol at 2-week intervals for 8 doses Group B: 6 nmol at 4-week intervals for 4 doses Group C: placebo	12–14 weeks	-TRSS (4 days of EEU challenge at 18–22 and 50–54 weeks post-treatment initiation) -Serum-specific IgE -AEs	-Group B had significantly better TRSS reduction compared to group A and the placebo at the 1-year follow up. -There were no changes in specific IgE levels compared to the baseline in any group. -No severe AE was reported in any group.
Couroux et al. (2015) [45]	RCT; Follow-up study of Patel et al. (2013) [44]	Cat allergen peptide (derived from Fel d 1)	Adults (*n* = 51)	Injection of allergen peptide at different intervals Group A: 3 nmol at 2-week intervals for 8 doses Group B: 6 nmol at 4-week intervals for 4 doses Group C: placebo	12–14 weeks	-TRSS (4 days of EEU challenge at 2 years post-treatment initiation) -AEs	-Group B demonstrated a trend toward reduction in mean TRSS compared to the placebo at 2 years after the EEU challenge; however, no statistical significance was shown. -Group A showed effects comparable to that of the placebo. -No severe AE was reported in any group.

Abbreviations: AEs, adverse events; EEU, environmental exposure unit; IDIT, intradermal immunotherapy; RCT, randomized controlled trial; SCIT, subcutaneous immunotherapy; TRSS, total rhinoconjunctivitis symptom score.
